# Detecting spatiotemporal clusters of accidental poisoning mortality among Texas counties, U.S., 1980 – 2001

**DOI:** 10.1186/1476-072X-3-25

**Published:** 2004-10-27

**Authors:** Ella T Nkhoma, Chiehwen Ed Hsu, Victoria I Hunt, Ann Marie Harris

**Affiliations:** 1 Department of Epidemiology, University of North Carolina at Chapel Hill, CB#7435, 2106-B McGavran-Greenberg Hall, Chapel Hill, NC 27599–7435, USA; 2 Department of Public and Community Health, University of Maryland College Park, 2371 HHP Building, Valley Drive, College Park, Maryland, 20742, USA; 3 Department of Health Management and Policy, University of North Texas Health Science Center, 3500 Camp Bowie Blvd, Fort Worth, Texas, 76107, USA; 4 Department of Environmental and Occupational Health, University of North Texas Health Science Center, 3500 Camp Bowie Blvd, Fort Worth, Texas, 76107, USA

## Abstract

**Background:**

Accidental poisoning is one of the leading causes of injury in the United States, second only to motor vehicle accidents. According to the Centers for Disease Control and Prevention, the rates of accidental poisoning mortality have been increasing in the past fourteen years nationally. In Texas, mortality rates from accidental poisoning have mirrored national trends, increasing linearly from 1981 to 2001. The purpose of this study was to determine if there are spatiotemporal clusters of accidental poisoning mortality among Texas counties, and if so, whether there are variations in clustering and risk according to gender and race/ethnicity. The Spatial Scan Statistic in combination with GIS software was used to identify potential clusters between 1980 and 2001 among Texas counties, and Poisson regression was used to evaluate risk differences.

**Results:**

Several significant (p < 0.05) accidental poisoning mortality clusters were identified in different regions of Texas. The geographic and temporal persistence of clusters was found to vary by racial group, gender, and race/gender combinations, and most of the clusters persisted into the present decade. Poisson regression revealed significant differences in risk according to race and gender. The Black population was found to be at greatest risk of accidental poisoning mortality relative to other race/ethnic groups (Relative Risk (RR) = 1.25, 95% Confidence Interval (CI) = 1.24 – 1.27), and the male population was found to be at elevated risk (RR = 2.47, 95% CI = 2.45 – 2.50) when the female population was used as a reference.

**Conclusion:**

The findings of the present study provide evidence for the existence of accidental poisoning mortality clusters in Texas, demonstrate the persistence of these clusters into the present decade, and show the spatiotemporal variations in risk and clustering of accidental poisoning deaths by gender and race/ethnicity. By quantifying disparities in accidental poisoning mortality by place, time and person, this study demonstrates the utility of the spatial scan statistic combined with GIS and regression methods in identifying priority areas for public health planning and resource allocation.

## Background

The accidental poisoning mortality rate has been increasing in the United States. In the 21-year period spanning 1981 to 2001, mortality rates due to accidental poisoning more than doubled from 2.0 per 100,000 in 1981 to 4.9 per 100,000 in 2001 [1]. The burden of accidental poisoning mortality in this period was more than four million years of potential life lost (YPLL) before age 65 [1]. Accidental poisoning mortality trends in Texas have mirrored national trends with a linear increase in rates from 1.5 per 100,000 in 1981 to 5.2 per 100,000 in 2001. During this period, 10,406 total deaths attributable to accidental poisoning have contributed to more than 250,000 YPLL before age 65 [1].

Accidental poisoning refers to the physiologic damage caused by inhalation, ingestion, or any other mode of exposure to various licit and illicit drugs, or chemicals such as those found in pesticides, household cleaning products and gases/vapors [2]. According to the US CDC classification standards, this category excludes poisonings that are associated with suicidal or homicidal intent. Several studies have examined geographic and temporal trends in accidental poisoning morbidity and mortality. Patterns of accidental poisoning have been shown to vary by gender, ethnicity, socioeconomic status, and region thus exhibiting non-random spatial or temporal distributions [3-5]. The study conducted by Hanson and Wieczorek [6] used the spatial scan statistic to identify spatial clusters of alcohol-related mortality. Altmayer et al. [7] reported significant disparities in premature mortality due to poisoning among certain geographic areas. An early study conducted in Ontario, Canada found spatial and temporal variation in accidental poisoning mortality rates, which also reported higher poisoning mortality in males than in females [8]. Kaufmann, Staes & Matte in their study on lead poisoning mortality reported elevated rates in less populated areas and different time trends in mortality by race and gender [9].

Many of the studies regarding poisoning mortality have examined exclusively excess mortality due to long-term exposure to specific agents and have not explicitly studied the possible persistence of mortality due to accidental poisoning across space and time. They often examined either spatial or temporal attributes of the burden of the disease. A statistic for examining spatial and temporal data concurrently is available through the use of SaTScan software. The objectives of this study are to detect the existence of spatial and temporal clusters of accidental poisoning mortality in Texas and to determine if there are variations with respect to race and gender among these clusters.

## Results

**Figure 1:**
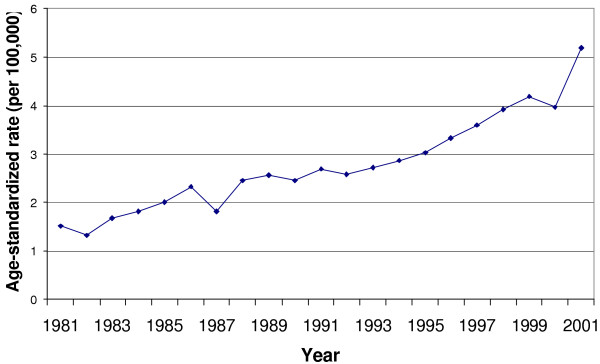
Accidental poisoning mortality trends in Texas, 1981 – 2001.

From 1980 to 2001, there were 10,774 deaths attributable to accidental poisoning, resulting in an average annual age-adjusted mortality rate of 2.8 deaths per 100 000. From 1981 to 2001, a linear increase was observed in unintentional poisoning mortality rates among Texas counties (annual percent increase = 1.06, 95% CI: 1.05 – 1.06, p < 0.0001, see Figure 1). Age-adjusted mortality rates were observed to be higher in men than in women, and to be highest among the Black population (see Table 1).

**Table 1:** Characteristics of study population, Texas, 1980 – 2001

**Population**	**Average Total Population (%)**	**Cumulative Deaths (%)**	**Annual age-adjusted rate (per 100,000)**
**All**	17,577,292 (100)	10,774 (100)	2.8
Male	8,677,605 (49.37)	7,593 (70.48)	4.0
Female	8,899,687 (50.63)	3,181 (29.60)	1.6
**White**	10,553,321 (60.04)	6,913 (64.16)	3.0
Male	5,187,210 (29.51)	4,661(43.26)	4.1
Female	5,366,111 (30.53)	2,252 (20.90)	1.9
**Black**	2,052,901 (11.68)	1,584 (14.70)	3.5
Male	991,066 (5.638)	1,063 (9.866)	4.9
Female	1,061,835 (6.041)	521 (4.836)	2.2
**Hispanic**	4,582,804 (26.07)	2,210 (20.51)	2.2
Male	2,305,930 (13.12)	1,820 (16.89)	3.6
Female	2,276,873 (12.95)	390 (3.620)	0.8
**Other**	388,266 (2.21)	67(0.62)	0.8
Male	193397 (1.10)	49 (0.45)	1.2
Female	194869 (1.11)	18 (0.17)	0.4

### Poisson regression results

After adjusting for age, regression analysis revealed significant associations between gender and race/ethnicity with accidental poisoning mortality. Males were found to be at higher risk of death from accidental poisoning than females (see Table 2). Among the race/ethnicity categories, the highest effect estimate (rate ratio) was for the Black population. The White population exhibited the second highest risk estimate, and the populations in the "other" category exhibited the lowest risk of accidental poisoning mortality (see Table 2).

**Table 2:** Rate ratios (RR) for accidental poisoning mortality using Poisson regression adjusted for age, gender, and race/ethnicity.

Factor	Rate Ratio	95% Confidence Interval	p-value
**Gender**			
Male	2.47	2.45 – 2.50	<0.0001
Female	1.00 (reference)	---	---
**Race/Ethnicity**			
White	1.00 (reference)	---	---
Black	1.25	1.24 – 1.27	<0.0001
Hispanic	0.79	0.78 – 0.80	<0.0001
Other	0.24	0.22 – 0.25	<0.0001

### Space-Time scan results

**Figure 2:**
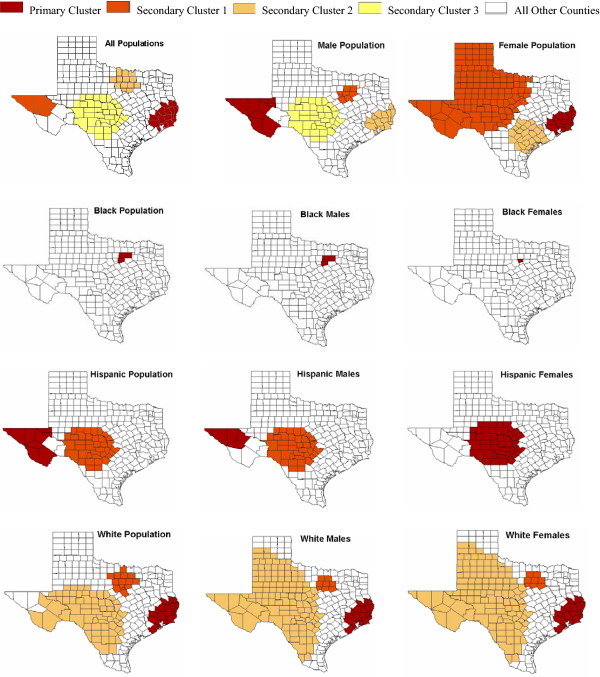
**Statistically significant accidental poisoning mortality clusters among Texas counties, 1980 – 2001. **See Table 3 for descriptive and effect information for each statistically significant cluster.

For the total population adjusting for age, gender and race/ethnicity, spatiotemporal scan analysis for accidental poisoning deaths among Texas counties revealed four significant clusters of high mortality rates (see Table 3). The most likely cluster was a "hotspot" cluster (i.e. relative risk > 2.0) [10] consisting of 16 counties located in the Gulf Coast and Southeast regions of Texas (see Figure 2). Another hotspot cluster was detected in the eastern portion of Texas and consisted of five counties. The primary cluster (i.e. most likely cluster) persisted for a shorter time period (four years) than the other clusters (see Table 3).

**Table 3:** Statistically significant spatiotemporal clusters of accidental poisoning mortality in Texas by gender and race/ethnicity

**Cluster**	**Time Period**	**No. of Observed Cases**	**Annual age-adjusted rate (per 100,000)**	**Relative Risk**	**p-value**
**All populations**					
Primary cluster	1998–2001	1095	5.7	2.056	0.001
Secondary cluster 1	1985–2001	637	6.8	2.448	0.001
Secondary cluster 2	1995–2001	1419	4.5	1.598	0.001
Secondary cluster 3	1995–2001	886	4.6	1.661	0.001
**All Males**					
Primary cluster	1985–2001	575	10.9	2.744	0.001
Secondary cluster 1	1995–2001	960	6.8	1.711	0.001
Secondary cluster 2	1998–2001	685	7.4	1.863	0.001
Secondary cluster 3	1994–2001	704	6.4	1.620	0.001
**All Females**					
Primary cluster	1997–2001	456	3.9	2.371	0.001
Secondary cluster 1	1996–2001	387	2.8	1.751	0.001
Secondary cluster 2	1997–2001	207	2.9	1.786	0.001
**White Population**					
Primary cluster	1998–2001	785	7.4	2.480	0.001
Secondary cluster 1	1996–2001	886	5.2	1.743	0.001
Secondary cluster 2	1995–2001	598	4.8	1.601	0.001
**White Males**					
Primary cluster	1998–2001	482	9.3	2.273	0.001
Secondary cluster 1	1995–2001	697	7.2	1.768	0.001
Secondary cluster 2	1994–2001	621	6.2	1.509	0.001
**White Females**					
Primary cluster	1998–2001	300	5.6	2.940	0.001
**Black Population**					
Primary cluster	1993–2001	345	6.3	1.800	0.001
**Black Males**					
Primary cluster	1993–2001	236	9.1	1.867	0.001
**Black Females**					
Primary cluster	1996–1998	2	2338.7	1048.700	0.033
**Hispanic Population**					
Primary cluster	1985–2001	467	6.0	2.730	0.001
Secondary cluster 1	1994–2001	342	3.8	1.730	0.001
**Hispanic Males**					
Primary cluster	1985–2001	410	11.2	3.130	0.001
Secondary cluster 1	1995–2001	239	6.0	1.663	0.001
**Hispanic Females**					
Primary cluster	1994–2001	84	1.8	2.264	0.001

The spatiotemporal scan statistic also revealed four significant clusters for the male population (see Table 3). The primary cluster for the male population was a hotspot cluster consisting of 10 counties located in East Texas. The second most likely cluster consisted of nine counties in North Central Texas (see Figure 2). For the male population, the primary cluster persists from 1985 into the present decade. The primary cluster for the female population closely mirrors that of the total population in location and size and persists from 1997 into the present decade. The second most likely cluster for the female population encompasses a large area of frontier (i.e. sparsely populated) counties in the eastern half of Texas (see Figure 2).

There were no real hotspot clusters observed for the Black population (see Table 3). An extreme effect estimate or fluctuation was observed for the Black female population due to the small number of observed deaths in the numerator. The number of cases was two and the period of persistence extended over two years. The significant cluster for the Black population was observed in three highly populated counties in North Texas (see Figure 2). This cluster persists for eight years and into the present decade.

A hotspot cluster was observed for the total Hispanic population consisting of ten counties in eastern Texas and it persisted for 16 years and entered into the present decade (see Figure 2 and Table 3). Five of the counties in the total Hispanic population cluster included a hotspot cluster for Hispanic males persisting for the same period of time. For Hispanic females, a hotspot cluster was observed in Central Texas consisting of 45 counties. This cluster persisted over a seven-year period beginning in 1994 and entering into the present decade (see Figure 2).

The non-Hispanic White population exhibited a hotspot cluster in the Gulf Coast region of Texas, consisting of seventeen counties. The cluster persisted for three years and into the present decade (see Table 3). The same spatiotemporal cluster was observed for the non-Hispanic White male population, albeit with a slightly different effect estimate. The most likely cluster for the non-Hispanic White female population was also a hotspot cluster. This cluster is located in the same region as the total non-Hispanic White population and persists for the same time period but only includes fifteen counties (see Figure 2).

No significant clusters were detected for populations in the "Other" category. There were very few accidental poisoning deaths for this population and the population count in many of the counties was zero for this subgroup. This resulted in instability in rates and other estimates for this population group (see Table 1).

### Spatial only and spatiotemporal scan adjusting for time non-parametrically results

**Figure 3:**
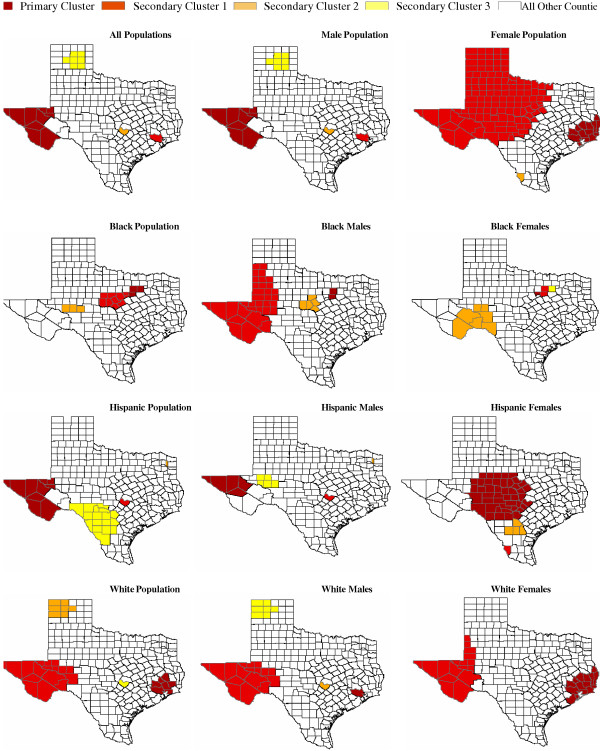
**Spatial accidental poisoning mortality clusters among Texas counties, 1980 – 2001. **Figure includes statistically significant and non statistically significant clusters to facilitate comparison with Figure 1. See Table 4 for descriptive and effect information for each statistically significant cluster.

**Figure 4:**
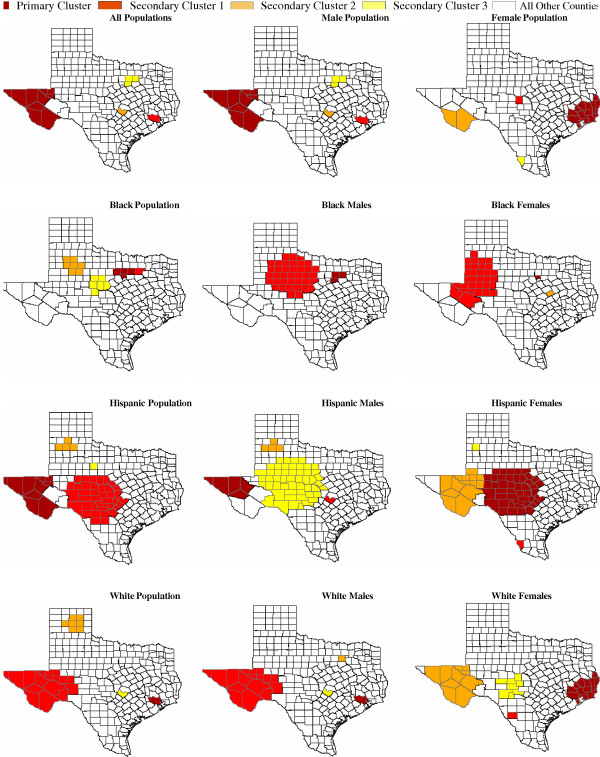
**Accidental poisoning mortality clusters among Texas counties adjusting for time nonparametrically, 1980 – 2001. **Figure includes statistically significant and nonstatistically significant clusters to facilitate comparison with Figure 1. See Table 5 for descriptive and effect information for each statistically significant cluster.

To verify whether the detected clusters using "spatial-temporal" methods are truly geographic clusters or space-time clusters not explained by the time trend, we further conducted two analyses using 1) the purely spatial scan statistic and 2) the space-time scan statistic non-parametrically adjusting for time. The results of these additional analyses indicated that most clusters detected using the default method were in mostly identical regions detected in the earlier analysis, only with slightly lower relative risks (i.e., observed deaths/expected deaths). For instance for the "all population group", the detected primary cluster in West Texas with a relative risk of 2.4 remained a cluster, but reduced to relative risks of 2.2 and 2.1 using additional methods that adjusted for time trend. In addition, most of the detected clusters still persisted to the present decade after the adjustments of time trend. Similar results were observed for most clusters detected in 'Male", "Female", "Black", "Non-Hispanic Whites" and "Hispanic" subpopulations using additional analyses options (see Tables 4 and 5). The summary maps of these additional analyses are presented in Figures 3 and 4.

**Table 4:** Statistically significant spatial clusters of accidental poisoning mortality in Texas by gender and race/ethnicity

**Cluster**	**No. of Observed Cases**	**Annual age-adjusted rate (per 100,000)**	**Relative Risk**	**p-value**
**All populations**				
Primary cluster	731	6	2.152	0.001
Secondary cluster 1	2408	3.6	1.285	0.001
Secondary cluster 2	520	3.7	1.326	0.001
Secondary cluster 3	148	4.1	1.468	0.011
Secondary Cluster 4	52	5	1.813	0.049
**All Males**				
Primary cluster	624	9.7	2.433	0.001
Secondary cluster 1	1685	5	1.269	0.001
Secondary cluster 2	404	5.7	1.434	0.001
**All Females**				
Primary cluster	962	2.1	1.278	0.001
**White Population**				
Primary cluster	1725	4	1.351	0.001
Secondary cluster 1	290	4.8	1.622	0.001
Secondary cluster 2	114	4.6	1.539	0.015
**White Males**				
Primary cluster	1030	5.7	1.399	0.001
Secondary cluster 1	217	7.3	1.782	0.001
Secondary cluster 2	269	5.8	1.414	0.001
**White Females**				
Primary cluster	704	2.6	1.337	0.001
**Black Population**				
Primary cluster	482	4.3	1.224	0.001
**Black Males**				
Primary cluster	108	7.2	1.476	0.039
**Black Females**				
Primary cluster	2	373.8	167.634	0.022
**Hispanic Population**				
Primary cluster	500	5.3	2.459	0.001
Secondary cluster 1	120	3.9	1.788	0.001
**Hispanic Males**				
Primary cluster	438	10	2.822	0.001
Secondary cluster 1	95	6	1.696	0.001
**Hispanic Females**				
Primary cluster	128	1.2	1.524	0.001

**Table 5:** Statistically significant spatiotemporal clusters of accidental poisoning mortality in Texas by gender and race/ethnicity, adjusting for time nonparametrically

**Cluster**	**Time Period**	**No. of Observed Cases**	**Annual age-adjusted rate (per 100,000)**	**Relative Risk**	**p-value**
**All populations**					
Primary cluster	1984–2001	654	6.3	2.261	0.001
Secondary cluster 1	1983–2001	2257	3.6	1.306	0.001
Secondary cluster 2	1990–1999	331	4.4	1.590	0.001
Secondary cluster 3	1993–1999	1088	3.5	1.264	0.001
Secondary cluster 4	1992–1994	38	8.9	3.207	0.002
**All Males**					
Primary cluster	1984–2001	588	10.0	2.521	0.001
Secondary cluster 1	1982–1993	797	5.9	1.475	0.001
Secondary cluster 2	1990–2001	337	6.4	1.605	0.001
Secondary cluster 3	1993–2001	1084	5.0	1.259	0.001
Secondary cluster 4	1992–1994	27	13.0	3.284	0.027
**All Females**					
Primary cluster	1983–2001	916	2.1	1.320	0.001
**White Population**					
Primary cluster	1983–2001	1390	4.3	1.439	0.001
Secondary cluster 1	1980–1997	220	5.5	1.832	0.001
Secondary cluster 2	1992–1994	32	11.0	3.679	0.001
Secondary cluster 3	1991–1999	207	4.6	1.536	0.003
Secondary cluster 4	1993–2001	544	3.7	1.246	0.019
**White Males**					
Primary cluster	1983–2001	950	5.9	1.439	0.001
Secondary cluster 1	1980–1998	179	8.1	1.987	0.001
Secondary cluster 2	1993–2001	416	5.8	1.421	0.001
Secondary cluster 3	1990–1999	171	6.8	1.653	0.001
Secondary cluster 4	1992–1994	24	16.5	1.037	0.004
**White Females**					
Primary cluster	1983–2001	638	2.7	1.409	0.001
**Black Population**					
Primary cluster	1993–2001	122	6.2	1.758	0.002
**Black Males**					
Primary cluster	1993–2001	236	7.2	1.477	0.001
**Black Females**					
Primary cluster	1996–1998	2	2031.6	911.05	0.026
**Hispanic Population**					
Primary cluster	1984–2001	477	5.6	2.592	0.001
Secondary cluster 1	1993–2001	368	3.2	1.483	0.001
**Hispanic Males**					
Primary cluster	1983–2001	424	10.4	2.923	0.001
Secondary cluster 1	1989–2001	88	7.1	2.005	0.001
**Hispanic Females**					
Primary cluster	1994–2001	84	1.5	1.920	0.002

## Discussion

The results from this study support the existence of spatiotemporal clusters of accidental poisoning mortality among Texas counties and show variations in these clusters by gender and race. This study also identifies several hotspot clusters. Moreover, the additional analyses (spatial only and adjusting for time non-parametrically) identify most clusters in almost identical regions but with slightly lower relative risks. This observation, along with the fact that both the results of the space-time scans with and without adjusting for time non-parametrically both demonstrate the temporal persistence of accidental poisoning mortality clusters into the present decade (at least 60% of the clusters persisted to the present decade using time-adjusting spatial scan), has provided supporting evidence that the clusters detected using the spatial-temporal method are geographic in nature, rather than an artefact of temporal trend.

The burden of excess accidental poisoning mortality was found to be highest in the Black population, followed by the non-Hispanic White population, and the least among the Hispanic population and populations of other race/ethnicity categories. Consistent with the literature, the male population also exhibited an elevated risk of accidental poisoning mortality when compared to the female population.

The results of this research presented both agreement and disagreement when compared other the results in which conventional techniques were used to identify geographic disparities by race/ethnicity, and gender. For instance, the results of this study were consistent with the literature in that the Black population was found to be at greatest risk of death from accidental poisoning [9,11]. In addition, the increased risk of accidental poisoning mortality observed in males when compared to females is consistent with what has been reported in other studies conducted in the United States [5,8,9,12]. On the other hand, the finding that the Hispanic population exhibited more than 20% less risk than the White population is unexpected. Other studies have found the Hispanic population to be either at increased risk or equal risk to the White population [5,11,13].

Although there are few studies in the literature that explicitly record cluster detection for accidental poisoning mortality, there are a variety of studies that have examined trends of accidental poisoning mortality in space and time [3-5,11,14]. Many of these studies have been conducted for specific toxic agents, e.g., alcohol, lead and pesticides but have not recorded the existence of spatial and temporal clustering of poisoning events and poisoning deaths.

There is also evidence in the literature to support the existence of temporal clustering of accidental poisoning mortality. In a study on organophosphate poisoning, Sahin, Sahin and Arabaci reported that deaths from accidental poisoning due to organophosphates were most frequent during certain months [4]. In a review of childhood poisoning, McGuigan reported temporal variations in symptomatic poisoning events over many years [14]. Sudakin, Horowitz, and Griffin used the space-time scan statistic to identify spatial and temporal clustering of accidental poisoning events due to pesticide exposures [15]. Therefore, the temporal clustering observed in the present study may be reflective of different types of poisoning agent exposures for each cluster.

The results of this study should be interpreted with several considerations. One, the process of cluster detection is necessarily ecological. The objective was to determine if there was an association between certain areas of Texas with excess accidental poisoning mortality. These results cannot be extrapolated to the level of the individual, i.e., one cannot interpret a relative risk above one as increased risk of accidental poisoning mortality for residents living in a given county. However, the risk estimate does provide valuable information about geographic disparity of accidental poisoning mortality.

Another consideration concerns the utilization of county-level data. The scan statistic may not have been sensitive to small areas of excess mortality that may have been detected at a higher geographic resolution. However, in Texas the county is the smallest geographic area for which there is routinely reported or available deaths and population estimate information. Also, the county is the political level at which health policy actions are instituted, since many of the local public health departments in Texas are county health departments.

A third consideration is that this study relied on county-level mortality data; and there may be variations in coding or reporting practices from county to county. Furthermore, in 1998 the International Classification of Diseases underwent its 10^th ^revision, and there may be differences in mortality estimates based on the codes in the 9^th ^and 10^th ^revisions. This introduces the possibility of misclassification bias in the results. However, there is no evidence to indicate that the misclassification would be differential [2]. Hence, any misclassification would bias the effect estimates towards the null, thus underestimating the actual effect. Thus, the results of this study still provide useful information concerning health disparities at the county, regional and state level.

## Conclusions

This study has demonstrated the existence of spatiotemporal clusters of accidental poisoning mortality in Texas. Additionally, this study has also shown variations in risk and clustering by gender and race/ethnicity. The results of this study have numerous implications. By quantifying accidental poisoning mortality disparities by geographic area, race/ethnicity, and gender, the results provide an evidence-base for health planning. The clusters identified in this study, specifically those that persist into the present decade, represent areas where health services, e.g., poison control centers and emergency rooms, may be deficient. Knowing the specific areas of excess mortality from accidental poisoning would help health policymakers to focus the scope of prevention programs and health care delivery, thus providing for efficient allocation of public health resources.

The findings from this study illustrate the use of the scan statistic and GIS in public health surveillance. Longitudinal data for accidental poisoning mortality was analyzed to identify hotspot clusters not only for the total population but also for subpopulations. Identifying geographic areas and specific populations, which are at an elevated risk of accidental poisoning mortality helps in the process of hypothesis generation for possible etiological mechanisms. For example, the most likely cluster for the total Texas population is in a heavily industrial area. The population in this area may have experienced excess mortality from other occupational exposure-related health outcomes (e.g. different types of cancers). One may further examine into the excess accidental poisoning mortality observed in this area in order to determine if it was due to occupational exposures.

The clusters identified in this study warrant further attention. Additional studies should be conducted to determine the causes behind the observed clustering, especially in the Gulf Coast region and the Central North Texas region where clustering was observed for the total Texas population and the two populations at greatest risk of accidental poisoning mortality, the Black population and the White population. More research is needed to assess different types of toxic exposures and their contribution to accidental poisoning mortality. Furthermore, the availability, locality and accessibility of health services such as poison control centers and health care facilities (hospitals and clinics) should be studied, giving special attention to those in non-urban counties.

## Methods

### Data sources and data processing

Three types of files were downloaded for the analysis. First, death files including the unintentional poisoning mortality data was obtained from Expert Health Data Programming Incorporated's Vitalweb (URL ). The data was extracted as counts by race, gender and age-group for ICD-9 codes 850 – 858 (unintentional poisoning by drugs) and 860–869 (accidental poisoning by solid, liquid, gas) for the years 1980 – 1998 and ICD-10 codes X40 – X49 (accidental poisoning by and exposure to noxious substances). The ICD-10 classification codes include exposure to drugs, gases and vapors, and other unspecified chemicals. Second, population (and estimates) files by year were obtained from the Texas State Data Center. Both deaths and population data included stratified information by age groups (16, representing the ages of 0–4 to 75 and above), gender (2, by male and female) and race (4, representing Blacks, Hispanics, Non-Hispanic Whites and Others). Third, county centroids information of latitude and longitude was downloaded from the 2000 U.S. Gazetteer portion of the US Census Bureau (Available online at ).

We performed the data processing by using an automated Microsoft Visual Basic Application (VBA) program to organize the downloaded county information of death counts, population at risk and geographic files (i.e. county centroids), before exporting them to SaTScan for analysis and for GIS mapping. The VBA program first imported, aligned, and then exported data tables to a SatScan compatible format. The program then invoked the SaTScan batch executable file (SaTScan version 4.0, freeware available from: URL: ) to perform scan analysis, and extracted cluster information from the SaTScan output file. Following this, the program linked the cluster dbase files with the county name file, and created a new file for mapping purposes. Using ArcGIS software (ESRI, ), we spatially joined the dbase file to a Texas county layer and produced maps displaying the poison mortality clusters of all populations and those in the populations stratified by gender, race/ethnicity, and gender-race/ethnicity combinations.

### Poisson regression

The association between gender, race/ethnicity, or the combination of both and accidental poisoning mortality was explored using Poisson regression. Poisson regression was conducted on accidental poisoning deaths using age, gender, year and race as predictor variables and the natural logarithm of population size as the offset variable. Regression analysis was conducted using the GENMOD procedure from the SAS system for Windows version 8.2 (Cary, North Carolina: SAS Institute, Inc.1999–2001). To control for overdispersion, the Poisson model was used with the scaled deviance option. Estimates of the annual percent increase, and rate ratios for race and gender were calculated from the results of the analysis.

### Spatial scan statistic

To detect potential spatiotemporal variations of poisoning mortality among Texas counties, this study utilized the Spatial Scan Statistic developed by Kulldorff [16,17] to detect clusters of unintentional poisoning mortality among Texas counties. This statistic has been used previously to detect clusters of breast cancer [18], and those specifically among Texas counties [19], prostate cancer [20], alcohol-related mortality [6], and diabetes prevalence [21] among others.

Many studies conducted on rare events such as unintentional poisoning deaths have aggregated the events to a higher geographic resolution, and have thus been unable to detect the existence of true clusters. The spatial scan statistic, however, is ideal for cluster detection of rare events since it includes the option of examining Poisson distributed data.

The space-time parameter of the spatial scan statistic places a cylindrical window on the coordinates grid for the locations studied and moves the center of the cylinder base over the grid so that the sets of geographic units covered by the window are constantly changing. The statistic assumes no predetermined cluster size and utilizes scan windows of various sizes (of the population at risk) with a maximum window size specified by the user. In the space-time scan, this spatial scan window serves as the base of a cylinder, with time acting as the height of the cylinder. The cylinder is continuously expanded and extended up to the maximum specifications set by the user. Whenever the cylindrical window finds a new death, SatScan calculates a likelihood function to test for elevated risk within the cylinder as compared with outside the cylinder. The likelihood function for any given cylindrical window (under the Poisson assumption) is proportional to:

(*d/n*)^*d*^([*D *- *d*] / [*D *- *n*])^(*D *- *d*)^*I*( )     (1)

where *D *is the total number of unintentional poisoning mortality deaths, *d *is the number of deaths within the space-time cylindrical window, and *n *is the expected number of cases after adjusting for any specified covariates. When SatScan is scanning for high rates, the indicator function *I*( ) is equal to 1 when the window has more cases than expected, and 0 if the observed cases are equal to or less than expected.

The present study utilized the retrospective space-time analysis for high rates using a Poisson model to calculate expected deaths due to unintentional poisoning in each county. The spatial scan window setting was set to a maximum cluster size of 25% of the study population, and the temporal scan window was set to a maximum cluster size of 90% of the study period. The covariates specified for this study were age group, gender, and race depending on the population being studied [16].

The "clusters" detected using the space-time scan statistic could be purely spatial, purely temporal or truly space-time clusters. To test whether these clusters are truly geographical in nature or are confounded by the temporal trend, i.e., whether there are any statistically significant geographical clusters or space-time clusters not explained by the time trend, we performed further analyses using (1) the purely spatial scan statistic and (2) the space-time scan statistic (non-parametrically) adjusting for time.

## Authors' contributions

ETN was responsible for the design and implementation of the project and the final paper. ETN also contributed to data analysis. CEH contributed to database design, visual basic application programming and data analysis. CEH also assisted in the preparation and editing of the final paper. VIH contributed to the preparation and editing of the final paper, including reviewing the relevant literature. AMH was responsible for the layout and design of the illustrative maps and also assisted in preparing and editing the final paper. All authors read and approved the final manuscript.
